# Cachectic Body Composition and Inflammatory Markers Portend a Poor Prognosis in Patients with Locally Advanced Pancreatic Cancer Treated with Chemoradiation

**DOI:** 10.3390/cancers11111655

**Published:** 2019-10-26

**Authors:** Patrick Naumann, Jonathan Eberlein, Benjamin Farnia, Jakob Liermann, Thilo Hackert, Jürgen Debus, Stephanie E. Combs

**Affiliations:** 1 Department of Radiation Oncology, University Hospital Heidelberg, Im Neuenheimer Feld 400, 69120 Heidelberg, Germany; 2 Clinical Cooperation Unit Radiation Oncology, dkfz Heidelberg, Im Neuenheimer Feld 280, 69120 Heidelberg, Germany; 3 Department of Radiation Oncology, University of Miami, 1475 NW 12th Avenue, Suite 1500, Miami, FL 33136, USA; 4 Department of General, Visceral and Transplantation Surgery, University Hospital Heidelberg, Im Neuenheimer Feld 110, 69120 Heidelberg, Germany; 5 Deutsches Konsortium für Translationale Krebsforschung (DKTK), Core Center Heidelberg, 69120 Heidelberg, Germany; 6 Department of Radiation Oncology, Technical University Munich (TUM), Ismaninger Straße 22, 81675 München, Germany; 7 Institute of Radiation Medicine (IRM), Department of Radiation Sciences (DRS), Helmholtz Zentrum München (HMGU), Ingolstädter Landstraße 1, 85764 Oberschleißheim, Germany; 8 Deutsches Konsortium für Translationale Krebsforschung (DKTK), Partner Site Munich, 81675 München, Germany

**Keywords:** locally advanced pancreatic cancer, chemoradiation, cachexia, weight loss, muscle wasting, adipose tissue, body composition, skeletal muscle index, sarcopenia

## Abstract

Background: Patients with pancreatic cancer often develop cancer cachexia, a complex multifactorial syndrome with weight loss, muscle wasting and adipose tissue depletion with systemic inflammation causing physical impairment. In patients with locally advanced pancreatic cancer (LAPC) neoadjuvant treatment is routinely performed to allow a subsequent resection. Herein, we assess body composition and laboratory markers for cancer cachexia both before and after neoadjuvant chemoradiation (CRT). Methods: Subcutaneous fat (SCF), visceral fat (VF), skeletal muscle (SM), weight and laboratory parameters were determined longitudinally in 141 LAPC patients treated with neoadjuvant CRT. Changes during CRT were statistically analyzed and correlated with outcome and Kaplan–Meier curves were plotted. Different prognostic factors linked to cachexia were assessed by uni- and multivariable cox proportional hazards models. Results: There was a significant decrease in weight as well as SCF, VF and SM during CRT. The laboratory parameter C-reactive protein (CRP) increased significantly, whereas there was a significant decrease in leukocyte count, hemoglobin, albumin and cholinesterase as well as in the tumor marker CA 19.9. Cachectic weight loss, sarcopenia, reductions in body compartments SCF, VF and SM, and changes in laboratory markers as well as resection affected survival in univariable analysis. In multivariable analysis, weight loss >5% (HR 2.8), reduction in SM >5% (HR 5.5), an increase in CRP (HR 2.2) or CA 19.9 (HR 1.9), and resection (HR 0.4) remained independently associated with survival, whereas classical cachexia and sarcopenia did not. Interestingly, the subgroup of patients with cachectic weight loss >5% or SM reduction >5% during CRT did not benefit from resection (median survival 12 vs. 27 months). Conclusions: Persistent weight loss and muscle depletion during CRT as well as systemic inflammation after CRT impacted survival more than cachexia or sarcopenia according classical definitions.

## 1. Introduction

Cancer cachexia is defined as a multifactorial syndrome with ongoing loss of skeletal mass, termed sarcopenia, leading to fatigue and physical impairment. An additional reduction of subcutaneous or intraabdominal fat compartments is not a prerequisite, but can often be observed [1,2]. Weight loss and adipose tissue wasting develop early and were specific for pancreatic tumor growth in mouse models [3]. Systemic inflammation with loss of appetite and a catabolic metabolism are thought to be among the driving factors. Cachectic weight loss and sarcopenia affect quality and length of life as well as response to oncologic treatments, such as surgery, chemotherapy and radiotherapy, particularly among patients with pancreatic cancer [4,5,6,7].

Pancreatic cancer is a severe disease that is often diagnosed late in advanced or even metastatic stages, resulting in five-year survival rates of 8% [8]. Surgical resection remains the only chance to achieve a cure, and consequently the resection margin is the most important predictor of survival [9,10]. Underpinning an often-late diagnosis and poor prognosis is the lack of early or specific symptomatology; patients usually present in advanced stages with icterus and cholestasis that occur when the tumor mass already blocks bile drainage and has encased the epigastric arteries, preventing primary surgical resection.

Nevertheless, neoadjuvant therapy consisting of chemotherapy and/or chemoradiation is routinely used in non-metastatic locally advanced pancreatic cancer (LAPC) that is initially deemed unresectable [11]. In selected patients who have a good response to neoadjuvant treatment and still no evidence of metastasis, surgical re-exploration with resection rates of 26% to 60% have been observed, which corresponds to more than a doubling of the median overall survival compared to unresected patients [12,13].

The decision to recommend surgery after neoadjuvant treatment is primarily based on patient performance status and tumor extent as seen using computer tomographic (CT) imaging. In addition, decision making at most centers is influenced by carbohydrate antigen 19.9 (CA 19.9) values. A decrease in CA 19.9 levels as a surrogate for response has been shown to correlate with successful resection despite interpretation as unresectable disease seen in imaging [14]. The prognostic importance of CA 19.9 was previously demonstrated in pancreatic cancer patients [15,16].

In terms of cachexia, other laboratory parameters may be important in selecting patients for surgery. For instance, inflammatory markers like tumor necrosis factor-alpha (TNF-α) and interleukin-6 (IL-6) are linked to cachexia and known to contribute to muscle loss and lipid wasting by increased protein breakdown and adipocyte lipolysis [17]. Yet, routine laboratory parameters, such as C-reactive protein, hemoglobin, albumin and cholinesterase, have also been implicated in patients with cachexia [18,19].

A 2015 review on cachexia and sarcopenia concluded that cachexia remains understudied in patients with pancreatic cancer [20]. Meanwhile studies examining sarcopenia in pancreatic cancer have substantially increased, but primarily address patients with resectable disease [4,5,6,21,22,23,24].

We previously reviewed evidence on the prognostic significance of continued weight loss and muscle depletion during CRT [25]; additional body composition markers such as subcutaneous fat, visceral fat, visceral obesity, sarcopenic visceral obesity as well as laboratory parameters associated with cachexia are the scope of the current retrospective analysis.

## 2. Results

In total, 141 patients met the inclusion criteria for assessment of tumor cachexia in LAPC. Patient characteristics are shown in Table 1.

All patients were treated with neoadjuvant concomitant chemoradiation (CRT) to a median dose of 54 Gy (range, 45–55 Gy) with once weekly gemcitabine 300 mg/^2^ body surface area (BSA). CRT lasted approximately 38 days and was followed by an additional cycle of gemcitabine (1000 mg/m^2^ BSA) until first follow-up. The average time from planning CT for treatment to a first follow-up CT to reassess resectability was 78.3 days, reflecting the study period for weight and body composition analysis.

### 2.1. Weight Loss and Changes in Body Composition

**Figure 1:**
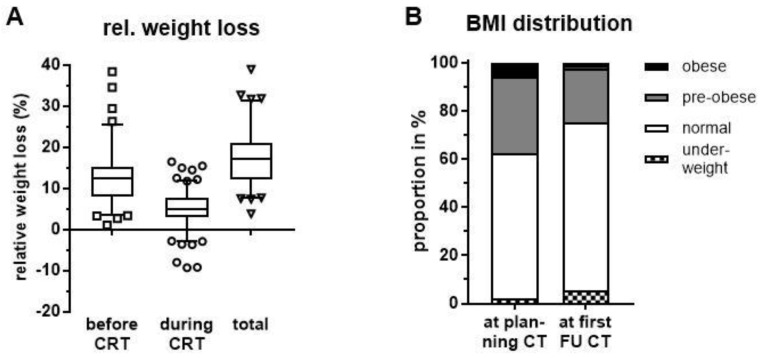
(**A**) Relative weight loss before initiation of chemoradiation (CRT) at planning computer tomography (CT), during CRT until first follow-up (FU) and in total. (**B**) BMI classification at planning CT before CRT initiation and after treatment completion at first FU CT.

Based on self-reported weight loss prior to CRT initiation, patients lost on average 12.8% (maximum 38.4%) of their body weight (Figure 1A). During the study period from the time of planning CT for radiation treatment to first follow-up (FU), there was a further mean relative weight loss of 5.3% (maximum 16.5%) which was statistically significant (*p* < 0.001, paired *t* test). Taken together, patients had a total relative weight loss of 17.6% (maximum 39.0%) at first FU when compared to initial weight before disease manifestation. Despite a majority of patients experiencing weight loss, 7 patients (5.0%) actually gained weight and 22 patients (15.6%) had stable weight during CRT; the former defined as an increase in weight by at least 2.4% and the latter as a change in weight <2.4% reflecting diurnal variation as previously described [26].

As a consequence of weight loss, there was a statistically significant drop in mean body mass index (BMI) from 24.2 kg/m^2^ to 22.8 kg/m^2^ during neoadjuvant CRT until first FU (*p* < 0.0001, Wilcoxon matched-pairs signed rank test). Consequently, there was an increase in patients classified as under-weight (BMI ≤ 18.5 kg/m^2^) at first FU. Similarly, the proportions of pre-obese (25 kg/m^2^ ≤ BMI < 30 kg/m^2^) and obese patients (BMI ≥ 30 kg/m^2^) declined considerably (Figure 1B).

To further characterize patients’ nutritional status, we assessed body composition longitudinally using CTs at two distinct time points: shortly before CRT at time of treatment planning and following treatment completion at first FU to re-assess resectability. Subcutaneous fat (SCF), visceral fat (VF) and skeletal muscle (SM) were segmented at the mid-plane of the third lumbar vertebra. To prevent possible measuring errors caused by patient positioning and gastrointestinal tract filling, we measured the whole volume from first (L1) to third lumbar vertebra (L3), rather than relying solely on the area from a single CT slice at L3.

**Figure 2:**
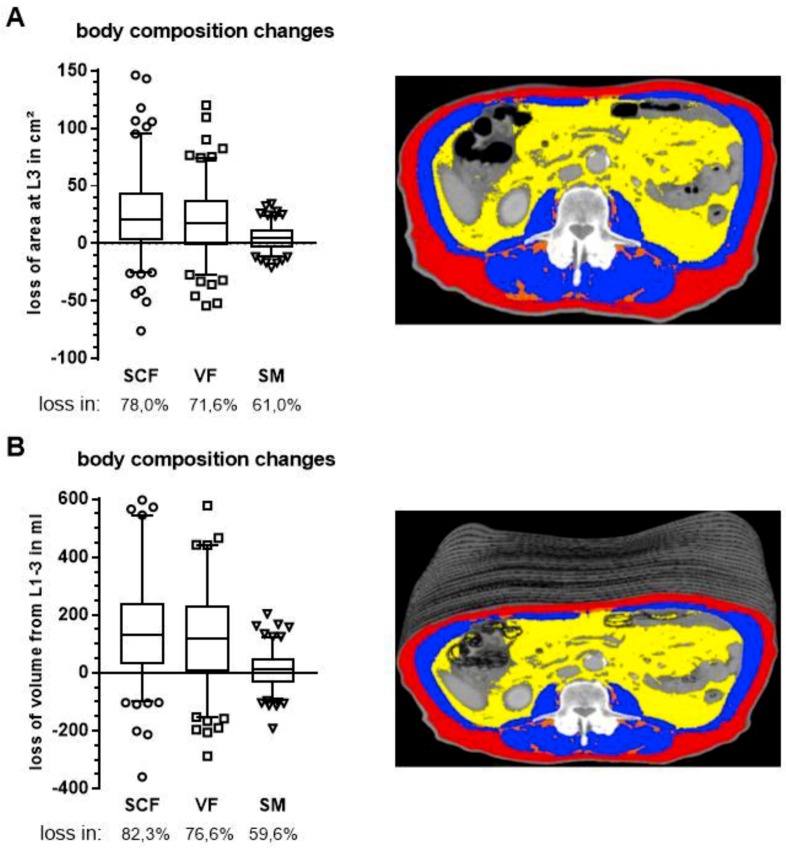
Changes in body composition during CT for radiation planning and CT for first FU as determined by segmentation of subcutaneous fat (SCF, red), visceral fat (VF, yellow) and skeletal muscle (SM, blue) in a single CT slice at third lumbar vertebra (**A**) and as volumes (**B**) of stacked CT slices from the first to third lumbar vertebra. Respective patient images appear on the right. Boxes extend from 25th to 75th percentiles and whiskers are drawn down to 5th percentiles and up to the 95th percentiles.

Congruent with patients’ weight loss, there was a statistically significant reduction in all three compartments: 142.1 cm^2^ to 115.2 cm^2^, 114.7 cm^2^ to 95.0 cm^2^ and 126.0 cm^2^ to 121.5 cm^2^ for SCF, VF and SM, respectively (*p* < 0.0001, Wilcoxon matched-pairs signed rank test, Figure 2A). Similarly, volumes of all three compartments were reduced from 778.6 mL to 620.9 mL, 749.6 mL to 618.3 mL and 772.9 mL to 758.9 mL over the segmented range for SCF, VF and SM, respectively (Figure 2B).

Changes in body composition occurred primarily in the fat compartments with 78.0% and 71.6% of patients having a decrease in SCF or VF, respectively. In contrast, muscle reduction occurred in only 61.0% of patients (Figure 2A). There was no difference between the single slice assessment at L3 and the volume assessment from L1 to L3.

Table 2 shows mean body composition and tumor size grouped by extent of surgical intervention (either resection or at least a surgical exploration) after neoadjuvant CRT, including those patients who were not surgical candidates.

A Kruskal–Wallis test followed by Dunn’s multiple comparison tests was performed to assess means but failed to identify any statistically significant differences between groups. Nevertheless, patients who underwent surgical intervention tended to have more SCF reserves and less loss of fat and muscle mass compared to those patients who were deemed unresectable after CRT. Moreover, patients who underwent resection had less VF before CRT initiation and a lower deficit at first FU. Interestingly, loss of skeletal muscle was quite balanced among the groups. As expected, tumor size after CRT tended to be smaller among those receiving surgical intervention compared to those who did not.

### 2.2. Cachexia Rates and Associated Blood Values before and after Neoadjuvant Chemoradiation

In accordance with international consensus, cancer cachexia is defined as either a relative weight loss >5% over 6 months in the absence of starvation, a BMI below 20 kg/m^2^ in combination with weight loss >2%, or sarcopenia in combination with weight loss >2% [27]. Sarcopenia is defined as depletion of muscles resulting in a skeletal muscle index (SMI) below sex specific cut-off values. The SMI is calculated by normalization of the SM area at L3 to the patient’s height.

Table 3 shows the proportions of weight loss, sarcopenia and cachexia utilizing respective definitions in the study population both before and after CRT.

At first FU, there was a statistically significant increase in patients with a relative weight loss >5% and a decrease in patients showing visceral obesity. As consequence, rates of sarcopenic visceral obesity (sarcopenia in combination with VF area >100 cm^2^) tended to be lower (*p* = 0.064) with a significant decrease of sarcopenic obesity (sarcopenia in combination with BMI ≥ 30 kg/m^2^) from 1.4% to 0.7% (*p* < 0.0001). While rates of sarcopenia increased at first FU, this was not statistically significant. Ultimately 90.8% of patients at first FU met cachexia criteria, a statistically significant increase compared to planning CT prior to CRT (*p* < 0.0001).

**Figure 3:**
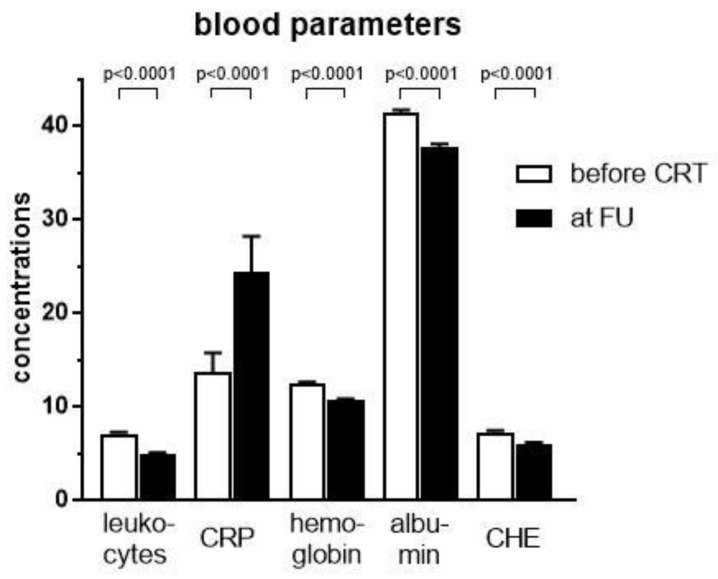
Laboratory variables including leukocytes (n/nL), CRP (C-reactive protein, mg/dL), hemoglobin (g/dL), albumin (g/L), and CHE (cholinesterase, kU/L) before and after neoadjuvant chemoradiation (CRT). Statistical differences were assessed using Wilcoxon matched-pairs rank test for all variables except hemoglobin, where a D’Agostino–Pearson normality test revealed a Gaussian distribution and a t-test for paired observations was therefore applied. Error bars indicate the standard error of the mean.

While there are no specific laboratory markers for cachexia, it is commonly agreed that inflammatory parameters, including leukocyte count, CRP, and CHE as well as hemoglobin and albumin are associated with cachexia [17,19]. Following treatment with neoadjuvant CRT, all parameters examined had significantly decreased, except CRP, where a statistically significant increase was observed (*p* < 0.0001, Figure 3).

To further characterize the impact of cachexia on inflammatory blood values, we assessed correlations with weight loss, skeletal muscle loss and resectability (Table 4).

Statistics revealed a positive correlation between the inflammatory marker C-reactive protein and weight loss as well as between leukocyte count and muscle loss. Albumin and cholinesterase negatively correlated for weight and muscle loss. Positive correlations for resectability included hemoglobin and cholinesterase values at first FU, indicating higher resection rates among patients with high hemoglobin and cholinesterase values post-treatment.

### 2.3. Survival Analysis

**Figure 4:**
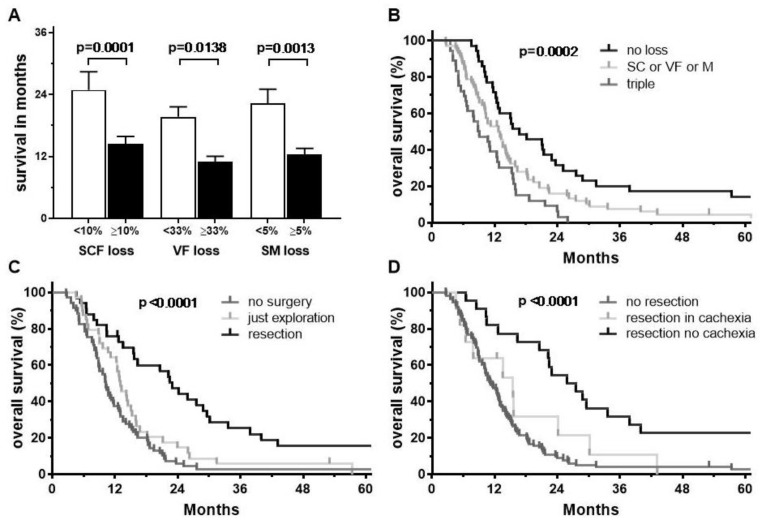
(**A**) Average survival according to amount of changes in body compartments with p-values of Mann Whitney test. Error bars indicate the standard error of the mean. (**B–D**) Kaplan–Meier survival curves grouped according to number of affected body compartments (**B**), surgery after CRT (**C**) and resection with or without cachectic weight and muscle loss (**D**).

Average survival following CRT was significantly different depending on the amount of abdominal tissue loss (Figure 4A). Patients with less than 10% loss of SCF had an average survival of 24.9 months compared to those who lost greater than 10% SCF, who had an average survival of 14.4 months (*p* = 0.0001). Similarly, patients with less than 5% reduction in skeletal muscle area had an average survival of 21.4 months compared to those who lost more than 5% (12.5 months, *p* = 0.0013). In terms of visceral fat area, differences were statistically significant when there was a reduction of at least one-third, with average survival of 11.2 months compared to 19.6 months among those who lost less than 33.3% (*p* = 0.0138).

After CRT, more than half of patients were deemed potentially resectable (*n* = 72) and 33 patients (23%) underwent a successful resection. Resections included reconstructions of vessels in 22 patients (67% of all resections) with involvement of arteries (*n* = 10), veins (*n* = 5) or both (*n* = 7). These patients had a median survival of 22.4 months and a 5-year overall survival rate of 16%. In contrast, patients who underwent surgical exploration with persistent unresectable disease or evidence of metastasis had a median survival of 12.8 months (*n* = 39, 28%). Similarly, those patients who were deemed unresectable at first follow-up or who refused to have surgery (*n* = 69, 49%) had a median survival of 10.2 months and a 5-year survival rate of 3% (Figure 4C). Among patients who underwent surgical resection, 11 (33%) experienced cachectic weight loss (at least 5%) or a reduction in skeletal muscle area of 5% or more during treatment. This subgroup had a median survival of 12.0 months compared to 26.7 months among the remaining resected patients (Figure 4D).

**Figure 5:**
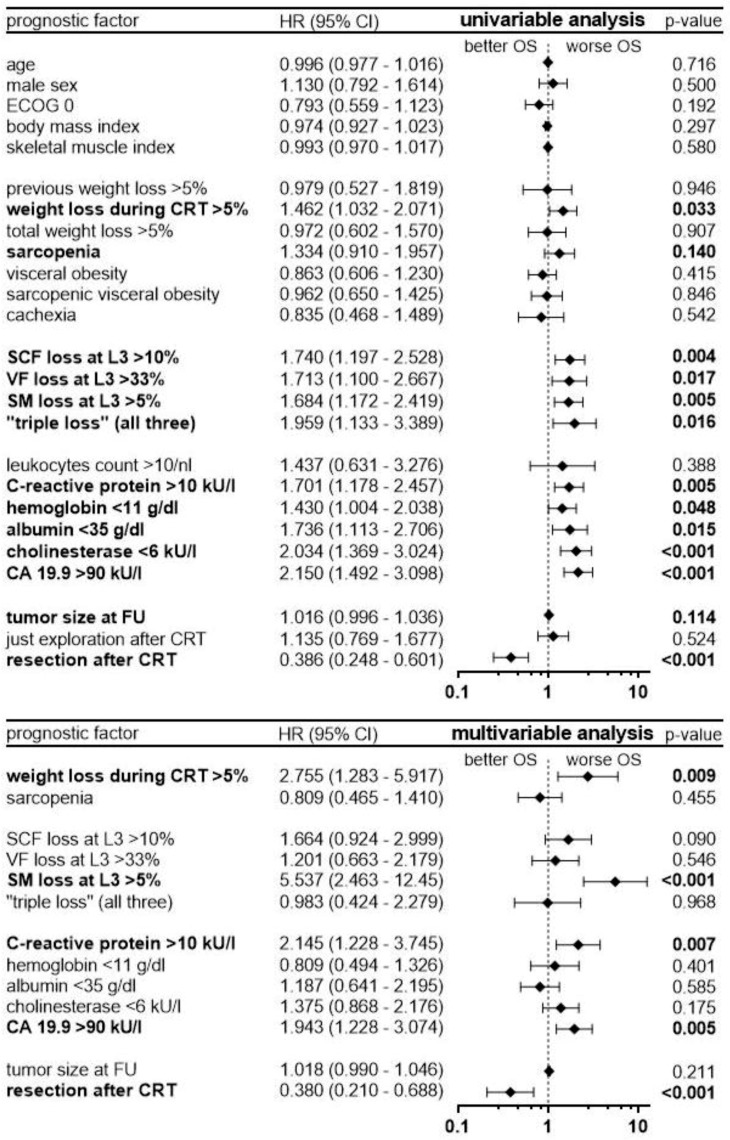
Uni- and multivariable Cox regression analysis with hazard ratios (HR) and 95% confidence intervals (CI). Factors with *p*-values < 0.15 in univariable cox regression were selected for multivariable analysis.

To evaluate hazard ratios (HR) of prognostic factors, uni- and multivariable Cox regression analysis were performed (Figure 5).

Univariable statistics identified the following prognostic factors as negative for survival: cachectic weight loss of at least 5%, sarcopenia and a reduction of SCF, VF, and SM by >10%, >33%, and >5%, respectively. A loss in all three compartments (“triple loss”) was a significant prognostic factor, too (HR 1.96, *p* = 0.016). Moreover, the laboratory parameters C-reactive protein values >10 kU/L, hemoglobin <11 g/dL, albumin <35 g/dL, cholinesterase <6 kU/L and CA 19.9 >90 kU/L as well as tumor size at first FU were prognostic factors associated with worse survival. Importantly, cachexia according to its classical definition had no impact on survival at first FU. Surgical resection after CRT was the most important prognostic factor for improved survival (HR 0.39, *p* < 0.0001).

In multivariable analysis, cachectic weight loss and ongoing muscle loss during CRT remained significant, independent prognostic factors, with resection after CRT remaining the most important factor (HR 0.38, *p* < 0.0001). Sarcopenia, as assessed by skeletal muscle index at first FU, and reductions in subcutaneous or visceral fat compartments were not statistically significant. Among the laboratory parameters, CA 19.9 and C-reactive protein remained significant independent, negative prognostic factors for survival.

## 3. Discussion

The present study assesses changes in weight, body composition and laboratory markers associated with tumor cachexia. The study population included a homogenous cohort of patients with unresectable, locally advanced pancreatic cancer consistently treated with neoadjuvant gemcitabine-based chemoradiation. At first FU, resectability was re-assessed and changes of the aforementioned variables analyzed for association with response and resectability as well as overall survival.

Compared to the time of CRT initiation, patients significantly lost body weight. This decrease was accompanied by significant reductions in the following body compartments in descending order: subcutaneous fat, visceral fat and skeletal muscle. Weight loss often occurs in patients with pancreatic cancer and since most patients had already lost weight before initiation of CRT, it is likely that CRT is not a de novo trigger. In fact, weight loss with muscle depletion and wasting of adipose tissue develop early and seem to be specific for pancreatic tumor growth, as reported in mouse models, but did not impair survival at least at this early stage [3]. Similarly, previous weight loss affected symptomatology and treatment side effects, but not survival as suggested in a series of patients with resectable pancreatic cancer [28]. Furthermore, weight loss was described in several studies of pancreatic cancer patients receiving mostly chemotherapy [21,29,30,31]. Moreover, the contribution of the small bowel to weight loss via the “microbiota-gut-brain axis” that might be disturbed by systemic treatments was also recently discussed [32].

Body composition changes predominantly occurred in the fat compartments with significant reductions in SCF, in line with previous reports [4,5,6,21,33]. Our volume measurements from L1 to L3 did not differ from single CT slice measurements at L3. Therefore, fat measurements in a single slice seemed not to be subject to differences in positioning and gastrointestinal filling as we previously expected. Changes in skeletal muscles occurred less frequently in our cohort, but impacted survival the most. As suggested before, muscle depletion occurs primarily in terminally cancer patients within a “90 days before death window” [34]. Our cohort had sarcopenia rates of 63% and 68% before and after CRT, respectively; these rates are at the upper end of published ranges of 30% to 67% among pancreatic cancer patients [20] and higher than 50% of a recent series of borderline resectable patients [30]. This might be explained by the fact that our cohort consisted of solely LAPC. On the other hand, in a large Japanese cohort of 265 patients and a large Italian cohort of 273 patients who all underwent curative surgery, rates of 64% and 65% were reported, respectively [5,23] supporting the idea that early muscle loss affects survival in patients with pancreatic cancer.

Sarcopenia occurs not only in slender or normal weight patients, but can affect over-weight patients, too; this so-called sarcopenic obesity was associated with poorer overall survival in pancreatic cancer and is defined when a BMI >30 kg/m^2^ occurs in combination with a low skeletal muscle index indicating sarcopenia [35,36]. Our cohort included only 8 obese patients with 2 having sarcopenic obesity before CRT initiation. Yet, we observed many patients with a surplus of visceral fat termed visceral obesity (>100 cm^2^ VF area at L3) that, when combined with sarcopenia or a high ratio of visceral fat to lean muscle area, is associated with major complications after resection and worse survival in pancreatic cancer [24,37]. As consequence of weight loss, we observed a decrease in patients having visceral obesity or sarcopenic visceral obesity from 56% and 34% to 40% and 27%, respectively.

Beyond muscle measurements for sarcopenia diagnosis, an impairment of muscle strength is an important feature of cachexia. Loss of muscle function can be explained by shrinkage and a fatty degeneration of muscles called myosteatosis that was found to correlate with worse outcomes in patients with pancreatic cancer [38]. Nevertheless, a recent study shows that myosteatosis is associated with high VF area, whereas sarcopenia is associated with low SCF area [39]. In our cohort, SM density was not altered during CRT, but due to threshold settings for muscle segmentation, the intramuscular fat was not included in SM area measurements. Interestingly another more pragmatic approach to quantify lean muscle was introduced as the psoas index, which is defined as the area of the psoas muscle at L3 normalized to the area of L3 itself. This index is determined faster than the SM area and was found to reliably predict for long-term survival in resectable pancreatic cancer [22]. To prevent muscle impairment, a recent clinical trial showed that progressive resistance training after resection of early stage pancreatic cancer is feasible and improves physical fitness [40]. The group with supervised training could even gain weight, whereas the control group had a stable weight.

Beyond an altered body composition, our cohort experienced a significant change in inflammatory markers as well as hemoglobin and albumin. While leukocyte counts and hemoglobin decreased most likely as a consequence of concomitant chemotherapy, there was an increase of C-reactive protein (CRP) and a decrease of albumin and cholinesterase. Given that pancreatic cancer itself fosters a microenvironment consisting of inflammatory cell infiltrates that enhances the immune system, it is hypothesized that cancer cachexia is triggered early too. Among others, tumor necrosis factor-alpha (TNF-α) and interleukin-6 (IL-6) and IL-8 were found to play a crucial role in cancer cachexia contributing to muscle loss and lipid wasting by increasing protein breakdown and adipocyte lipolysis [17,41,42]. Unfortunately, both parameters could not be assessed in our retrospective analysis since their levels were not available in routine care. In contrast, levels of the blood parameters CRP, hemoglobin, albumin and cholinesterase (CHE) were routinely determined. Concerning these parameters, an increase in CRP and reductions in hemoglobin and albumin as well as CHE were previously linked to cachexia [18,19]. In addition, high neutrophil to lymphocyte ratios and low albumin levels were correlated with muscle loss and myosteatosis in colorectal cancer patients and an association with systemic inflammatory response highlighted [43]. In a series of recurrent pancreatic cancer, low levels of CHE as well as anemia and hypoalbuminemia were identified as negative prognostic factors [44]. In our cohort weight loss was positively correlated with CRP and negatively correlated with albumin and CHE changes, whereas muscle loss was primarily negatively correlated with CHE changes. Resectability was associated with high hemoglobin and CHE levels, indicating importance of these values. In cox regression analysis, all laboratory parameters except leukocyte count influenced survival, but in final multivariable analysis only elevated CRP and CA 19.9 remained independent negative prognostic factors.

Following CRT more than half of patients were deemed resectable and surgery was successfully completed as resection in 23% (46% resection rate among operated patients). Resection was identified as the most important and independent positive prognostic factor of survival in cox regression analysis (HR 0.38). Patients who underwent only surgical exploration had a reduced survival, similar to patients who were deemed unresectable. Continued weight loss (HR 2.8) and muscle loss during CRT (5.5) were further independent prognostic factors for worse survival in multivariable analysis, whereas loss of adipose tissue was not, as recently reported for advanced pancreatic cancer [45]. In fact, only a subgroup with very high reductions of VF (>33%) showed reduced survival in our cohort, at least in Mann–Whitney U-Test and univariable cox-regression. Nevertheless, survival curves separate significantly in Kaplan–Meier analysis when grouped by no compartment reduction, a “triple” loss of SCF, VF and SM, or any other loss. Yet, given that this failed in multivariable analysis, the survival in this subgroup could be explained as being caused by an extensive weight loss itself. High rates of intrabdominal fat were reported to predict worse survival in resectable pancreatic cancer [46], but visceral obesity did not influence outcomes in our cohort as in another large cohort of 328 patients with resectable pancreatic cancer [47].

Interestingly, both sarcopenia, as determined by skeletal muscle index, as well as cachexia, as determined according to international consensus as weight loss within the last 6 months, or combination of sarcopenia and weight loss, or low BMI and weight loss [27,35], failed to predict for overall survival in multivariable analysis. An explanation might be that some patients gained weight and muscle mass during CRT that contributed to an improved survival, but this gain was not enough to change the status of having sarcopenia or cachexia. In fact, it seems that the actual dynamics of weight and especially muscle loss in a roughly 3-month period are more important for survival prediction, than classification as cachectic according to weight loss within 6 months or as sarcopenic according to a fixed muscle index. As a matter of fact, patients who lost >5% in body weight or muscle mass during CRT had the lowest survival irrespective of a successful resection.

Despite the limitation of the retrospective design of our study that is per se subject to bias, we see several strengths such as high patient numbers, homogenously treated cohort and longitudinal body composition measurements, incorporating the commonly used L3 level and volumes of L1 to L3, and measurements at comparable distinct therapeutic time points. However, prospective trials are needed to validate the data.

## 4. Materials and Methods

### 4.1. Patient Selection and Treatment Approach

We retrospectively enrolled 141 consecutive patients with LAPC referred to our department with unresectable LAPC for neoadjuvant treatment between 2007 and 2014. Inclusion criteria were initially unresectable disease, complete patient charts and available imaging to assess weight, body compartments and blood values shortly before treatment at time of planning CT and afterward at first follow-up. Histologic confirmation was required for all patients. However, if the biopsy results were not conclusive and a repeat biopsy was not possible or denied by the patient, LAPC status was determined based on interdisciplinary tumor board recommendations, integrating radiologic imaging and CA 19.9 levels characteristic of LAPC. Resectability was assessed according to NCCN criteria. Patients with stage IV disease prior to initiation of neoadjuvant CRT and who died of complications after surgery or hospital-acquired infection were excluded. CRT was delivered with photons utilizing conventional fractionation combined with once weekly gemcitabine 300 mg/m^2^ BSA, followed by one cycle of gemcitabine 1000 mg/m^2^ BSA until first follow-up for the re-evaluation of resectability. The study was approved by our institutional ethics committee (S-483/2011 and S-063/2019 according new data protection regulations).

### 4.2. CT Analysis, Sarcopenia, and Cachexia

Areas of subcutaneous fat (SCF), visceral fat (VF) and skeletal muscle (SM) were determined at the mid plane of the third lumbar vertebra using two CTs at two distinct time points for each patient: the first for planning simulation before treatment initiation and the second at first follow-up after treatment completion. Analysis was conducted using SliceOmatic^®^ 5.0 (Tomovison^®^, Magog, J1X 0R4, Canada). As previously reported, the third vertebral level is considered the best reference site to assess fat distribution and lean muscle [48] and was recently confirmed to have a good correlation with other established methods, including bioelectrical impedance analysis and dual x-ray absorptiometry [49]. At time of study conceptualization, we decided to perform volume measurements to compensate for possible measuring errors in the fat area caused by differences in patient positioning and gastrointestinal tract filling between two timepoints. Furthermore, a volumetric approach was considered superior to single slice measurements at that time [50]. Therefore, we determined the areas of SCF, VF and SM slice by slice from first to third lumbar vertebra for each patient. Each segmented area was then multiplied by its CT slice thickness (cm^2^ × mm) and divided by 10 to obtain the corresponding ml (cm^3^). If the CT for planning and that for first follow-up differed in slice spacing, the last slice was multiplied with an appropriate thickness to obtain equal scan heights (sum of spacing between first and last measured slice) in both CTs. This approach ensured that calculated volumes for both CTs were comparable.

Thresholds for measurements were set to −30 to −170 HU, −30 to −190 HU, −29 to +150 HU for SCF, VF and SM, respectively, as previously reported [34,51]. The resulting areas were manually checked and corrected by two independent physicians. Final areas comprised the subcutaneous fat (without skin), the visceral fat (whole intraabdominal fat without intra-intestinal fatty content) and muscles of the abdominal wall, the back and lumbar area, including the rectus abdominis, external oblique, transversus abdominis, internal oblique, psoas, quadratus lumborum, and erector spinae muscles. Due to threshold settings, intramuscular fat was excluded, but the corresponding area was measured separately. As previously reported, visceral obesity was defined as having a VF area of at least 100 cm^2^ [24]. The skeletal muscle index (SMI) was calculated for each patient by normalizing the measured SM area to the square of the patient’s height. As previously suggested, sarcopenia was defined when the SMI was below the sex-specific cut-off values of 38.5 and 52.4 cm^2^/m^2^ for females and males, respectively [35]. Sarcopenic visceral obesity was defined as the combination of both visceral obesity and sarcopenia. Finally, according to international consensus, cancer cachexia was defined as either a weight loss of greater than 5% over 6 months in absence of starvation, a BMI below 20 kg/m^2^ in combination with a weight loss >2%, or sarcopenia in combination with a weight loss >2% [27].

### 4.3. Statistical Analysis

Prism 7.04 software (GraphPad, San Diego, CA, USA) and SPSS Statistics Subscription (International Business Machines Corporation, Armonk, New York, NY, USA) were used to perform statistical analysis and graphical plotting of results. Results were considered significant when the *p*-value was less than 0.05. D’Agostino and Pearson omnibus K2 normality tests suggested a Gaussian distribution for the continuous variables of weight loss and the laboratory parameter hemoglobin and therefore paired *t-*tests were used to compare means of groups for statistical difference. Areas and volumes of SCF, VF and SMA were not distributed via Gaussian modeling, requiring the use of Wilcoxon matched-pairs signed rank tests. To compare the changes in body composition according to extent of surgical resection after CRT (no surgery, just surgical exploration, or resection), a Kruskal–Wallis test followed by Dunn’s multiple comparison tests as post-tests was applied as D’Agostino and Pearson omnibus K2 normality tests failed to identify a Gaussian distribution for each of the three grouping variables. The categorical variables of cachectic weight loss (>5%), sarcopenia, visceral obesity, sarcopenic visceral obesity and cachexia as well as the laboratory parameters of leukocyte count, C-reactive protein, albumin and cholinesterase did not follow Gaussian distribution and changes during CRT were analyzed by Wilcoxon matched-pair analysis. Corresponding correlations to weight loss, muscle loss and possible resectability after CRT were assessed by calculation of the non-parametric Spearman correlation coefficient *r*. The parametric Pearson correlation coefficient *r* was only applied for the laboratory parameter hemoglobin.

Overall survival was calculated using Kaplan–Meier and log-rank tests. Average overall survival in different groups were compared using non-parametric Mann–Whitney tests since D’Agostino and Pearson omnibus K2 normality tests revealed that overall survival rates did not follow a Gaussian distribution. Finally, a Cox regression analysis was performed assuming proportional hazards. A univariable model was first utilized to identify possible prognostic factors for overall survival, with promising variables (*p* < 0.15) subsequently selected for a multiple Cox regression analysis to test the overall model. Hazard ratios corresponding to the 95% confidence interval were calculated.

## 5. Conclusions

In conclusion, achieving a complete surgical resection after neoadjuvant treatment is crucial for prolonged survival in patients with locally advanced pancreatic cancer. Yet, surgery has less of an impact on survival if cachectic weight loss and muscle depletion continue during neoadjuvant CRT. Therefore, consideration of these variables along with the tumor marker CA 19.9 is paramount for the decision of whether to continue with surgery. Elevated values of C-reactive protein without a clinical focus of inflammation may indicate cachexia and a worse outcome, whereas changes in adipose tissue, sarcopenia and cachexia alone were not of prognostic significance. Future clinical trials should address whether early nutritional counseling aimed at preventing loss of weight and muscle mass may contribute to an increase in resection rates.

## Figures and Tables

**Table 1:** Demographic, baseline parameters and treatment duration.

Characteristics	Mean
Age, in years (SD)	63.6 (± 9.2)
Gender, *n* (%)	
Male	77 (54.6%)
Female	64 (45.4%)
Tumor Site, *n* (%)	
Head	73 (51.8%)
Body	22 (15.6%)
Tail	2 (1.4%)
Multiple	44 (31.2%)
Tumor Stage, *n* (%)	
I	0 (0%)
II	0 (0%)
III	141 (100%)
IV	0 (0%)
ECOG score, *n* (%)	
0	72 (51.1%)
1	58 (41.1%)
2	11 (7.8%)
3	0 (0%)
Prior chemotherapy	
None	126 (85.8%)
Gemcitabine	8 (5.0%)
Gemcitabine + Erlotinib	5 (3.6%)
Gemcitabine + Cisplatin	1 (0.7%)
Capecitabine	1 (0.7%)
FOLFIRINOX	3 (2.1%)
Unknown	3 (2.1%)
Height, in cm	170 (± 9.1)
Weight, in kg (SD; range)	70.1 (± 12.3; 44–115))
CA 19.9, in kU/L (SD; range; median)	1,286.5 (± 3,272; 0.1–27,031; 230.3)
Timeline, in days (SD)	
Planning CT to treatment initiation	9.0 (± 6)
Treatment duration	38.3 (± 4)
Treatment completion to follow-up CT	29.4 (± 9)
Planning CT to follow-up CT	78.3 (± 11)

CA: carbohydrate antigen, CT: computer tomogram, ECOG: Eastern Cooperative Oncology Group, FOLFIRINOX: folinic acid, fluorouracil, irinotecan and oxaliplatin.

**Table 2:** Changes in body composition and tumor size by extent of surgical intervention after chemoradiation.

Parameter	No Surgery (*n* = 69)	Surgical Exploration (*n* = 39)	Surgical Resection (*n* = 33)
	Mean (95% CI)	Mean (95% CI)	Mean (95% CI)
SCF area in cm^2^			
—before CRT	139.3 (119.9–158.7)	147.3 (123.0–171.5)	141.7 (111.1–172.4)
—at first FU	107.0 (92.4–121.7)	122.4 (103.7–141.1)	123.9 (94.5–153.3)
—deficit	32.3 (22.9–41.6)	24.8 (13.0–36.6)	17.8 (6.7–29.0)
VF area in cm^2^			
—before CRT	113.6 (95.8–131.5)	131.9 (110.9–152.9)	96.7 (75.6–117.8)
—at first FU	93.0 (79.6–106.4)	109.3 (94.2–124.4)	82.4 (62.5–102.4)
—deficit	20.6 (12.9–28.4)	22.6 (11.9–33.3)	14.2 (5.1–23.4)
SM area in cm^2^			
—before CRT	124.4 (118.5–130.2)	131.7 (120.6–142.8)	122.8 (112.8–132.9)
—at first FU	119.6 (114.1–125.0)	127.4 (116.7–138.2)	118.6 (109.8–127.4)
—deficit	4.8 (2.0–7.6)	4.3 (1.4–7.2)	4.2 (0.4–8.1)
Tumor size in cm			
—before CRT	5.0 × 3.7 (4.8−5.3 × 3.5−4.0)	4.7 × 3.4 (4.3−5.2 × 3.0−3.7)	4.6 × 3.5 (4.1−5.2 × 3.1−4.0)
—at first FU	4.8 × 3.5 (4.5−5.1 × 3.2−3.7)	4.4 × 3.2 (4.0−4.8 × 2.9−3.5)	4.2 × 3.2 (3.7−4.6 × 2.9−3.6)

**Table 3:** Proportions of patients fulfilling different body composition definitions.

Parameter	Before CRT at Planning CT *n* (%)	After CRT at CT for First FU *n* (%)	*p*-Value †
Cachectic weight (loss >5%)	83 (58.9%)	120 (85.1%)	**<0.0001**
Sarcopenia (SMI below cut-offs)	89 (63.1%)	96 (68.1%)	0.1892
Visceral obesity (VF at L3 >100 cm^2^)	79 (56.0%)	57 (40.4%)	**<0.0001**
Sarcopenic visceral obesity	48 (34.0%)	38 (27.0%)	0.0639
Sarcopenic obesity	2 (1.4%)	1 (0.7%)	**<0.0001**
Cachexia by definition	89 (63.1%)	128 (90.8%)	**<0.0001**

† Wilcoxon matched-pairs signed rank test.

**Table 4:** Laboratory values and correlation with weight loss, skeletal muscle loss, and resectability.

Parameter	Before CRT at Planning CT Mean (95% CI)	After CRT at CT for First FU Mean (95% CI)	Correlation Coefficient to Weight Loss	Correlation Coefficient to Muscle Loss	Correlation Coefficient to Resectability
Leukocyte, n/nL	7.1 (6.7–7.5)	4.9 (4.4–5.3)	0.120 † (*p* = 0.16)	0.216 † (*p* = 0.01)	0.032 † (*p* = 0.71)
C-reactive protein, mg/L	13.7 (9.7–17.7)	24.4 (17.0–31.9)	0.260 † (*p* = 0.002)	0.096 † (*p* = 0.26)	−0.139 † (*p* = 0.10)
Hemoglobin, g/dL	12.5 (12.2–12.7)	10.7 (10.5–10.9)	−0.025 * (*p* = 0.77)	0.089 * (*p* = 0.29)	0.209 * (*p* = 0.01)
Albumin, g/L	41.4 (40.9–42.0)	37.7 (36.9–38.5)	−0.206 † (*p* = 0.02)	0.085 † (*p* = 0.35)	0.023 † (*p* = 0.81)
Cholinesterase, kU/L	7.3 (7.0–7.6)	6.0 (5.6–6.3)	−0.187 † (*p* = 0.04)	−0.220 † (*p* = 0.02)	0.188 † (*p* = 0.03)

† non-parametric Spearman correlation coefficient of difference before and after CRT; * parametric Pearson correlation coefficient of difference before and after CRT.
